# The Beneficial Effects of a N-(1-Carbamoyl-2-phenyl-ethyl) Butyramide on Human Keratinocytes

**DOI:** 10.3390/ph18040517

**Published:** 2025-04-01

**Authors:** Franca Oglio, Serena Coppola, Alessandra Agizza, Antonio Masino, Chiara Luongo, Roberta Di Santillo, Ludovica D’Auria, Roberto Russo, Ilaria Neri, Lucia Grumetto, Laura Carucci, Erika Caldaria, Rita Nocerino, Ritamaria Di Lorenzo, Antonio Calignano, Sonia Laneri, Lorella Paparo, Roberto Berni Canani

**Affiliations:** 1 Department of Translational Medical Science, University of Naples Federico II, 80131 Naples, Italy; franca.oglio@unina.it (F.O.); serena.coppola3@unina.it (S.C.); agizza.alessandra@gmail.com (A.A.); antonio.masino@unina.it (A.M.); chialu86@gmail.com (C.L.); robertadisantillo@gmail.com (R.D.S.); laura.carucci@unina.it (L.C.); erikacaldaria@gmail.com (E.C.); rita.nocerino@unina.it (R.N.); 2 NutriTechLab at CEINGE Advanced Biotechnologies, University of Naples Federico II, 80131 Naples, Italy; paparolorella@gmail.com; 3 ImmunoNutritionLab at CEINGE Advanced Biotechnologies, University of Naples Federico II, 80131 Naples, Italy; 4 Ceinge Advanced Biotechnologies, University of Naples Federico II, 80131 Naples, Italy; 5 Department of Pharmacy, University of Naples Federico II, 80131 Naples, Italy; roberto.russo@unina.it (R.R.); ilaria.neri@unina.it (I.N.); lucia.grumetto@unina.it (L.G.); ritamaria.dilorenzo@unina.it (R.D.L.); calignan@unina.it (A.C.); sonia.laneri@unina.it (S.L.); 6 Department of Biomedicine and Prevention, University of Rome “Tor Vergata”, 00133 Rome, Italy; 7 Department of Laboratory Medicine, ASL Benevento, 82100 Benevento, Italy; 8 European Laboratory for the Investigation of Food-Induced Diseases, University of Naples Federico II, 80131 Naples, Italy; 9 Task Force for Microbiome Studies, University of Naples Federico II, 80131 Naples, Italy

**Keywords:** butyrate, skin microbiome, skin barrier, oxidative stress, keratinocyte proliferation, wound healing

## Abstract

**Background:** The skin microbiota-derived metabolite butyrate plays a pivotal role in maintaining skin health. Unfortunately, unpleasant sensorial properties and unfavorable physicochemical properties strongly limit the butyrate use in dermatology clinical practice. This study investigates the effects of N-(1-carbamoyl-2-phenyl-ethyl) butyramide (FBA), a butyric acid releaser with neutral sensorial properties on skin keratinocyte function. **Methods:** Immortalized human keratinocyte cell line (HaCaT cells) was treated with FBA at various concentrations (0.001–1 mM) and time points (6–48 h). Cellular proliferation was assessed using MTT assays, while barrier integrity was evaluated by measuring tight junction proteins (occludin and ZO-1). Oxidative stress was analyzed using ROS assays and Western blot for Nrf2 and NF-κB expression. Markers of differentiation and extracellular matrix proteins were measured via quantitative PCR and wound-healing capability was assessed using a scratch assay. **Results:** FBA significantly enhanced keratinocyte proliferation at an optimal concentration of 0.1 mM. Tight junction protein expression increased, indicating improved barrier function. FBA reduced oxidative stress by upregulating Nrf2 and suppressing NF-κB activity. It also promoted the expression of differentiation markers (e.g., keratin-1, filaggrin) and extracellular matrix proteins (e.g., collagen type I and elastin). Furthermore, FBA accelerated wound closure, demonstrating its efficacy in enhancing the mechanisms of skin repair. **Conclusions:** Our results demonstrate that FBA enhances human keratinocyte cell differentiation, proliferation, and skin repair while protecting against oxidative stress. Its potential in cosmetics lies in delivering butyric acid benefits without organoleptic limitations, with possible applications in several skin condition characterized by deficient butyrate production and inflammation, such as atopic dermatitis.

## 1. Introduction

Skin, the largest human organ, hosts a diverse and complex ecosystem inhabited by a high diversity of microorganisms referred to as the skin microbiome [1]. The skin microbiome plays a vital role in maintaining skin health and well-being [2]. Many of the microbiome effects on skin health are derived from the activity of metabolites [3]. Among these metabolites, butyrate emerged as one of the most relevant players in modulating skin health [3,4,5]. Butyrate is a short-chain fatty acid with benefits for skin health, including anti-inflammatory, barrier-enhancing, wound-healing, antimicrobial, antioxidant, anti-redness, and lightening properties [3,6]. These features support the use of butyrate in cosmetics. Unfortunately, unfavorable sensorial and physicochemical properties strongly limit the dermatologic use of butyrate [6,7]. In fact, butyrate has an unpleasant odor, high volatility, and water solubility, leading to rapid dissociation and reduced bioavailability at physiological pH. This limits its stability in topical formulations, potentially affecting its therapeutic efficacy [8]. Previous studies addressed these limitations by exploring how to improve butyrate stability, usability, and acceptability in dermatological applications [8]. For instance, butyrate esters phenylbutyrate are more stable and odorless but requires enzymatic conversion to release active butyrate, or tributyrin, a triglyceride form that provides controlled butyrate release while reducing odor [9]. Also, there are encapsulation techniques to reduce volatility, to mask odor, and to enhance stability.

A butyrate releaser, N-(1-carbamoyl-2-phenyl-ethyl) butyramide (FBA), has been recently proposed as a valid alternative for the topical use of butyrate in the dermatological and cosmetic fields. It rapidly releases butyric acid, has no smell, and shares the same pharmacokinetic and safety profiles of this short-chain fatty acid [7]. This compound has gained attention in recent years for its potential therapeutic applications, particularly due to its enhanced stability and bioavailability compared to natural butyrate [6,7].

In this context, it has been recently demonstrated that FBA holds soothing and anti-redness properties for human skin [7,9]. In a clinical study involving twenty healthy female volunteers, it has been shown that applying an emulsion containing FBA significantly reduced skin redness and the erythema index caused by induced erythema using a high concentration of SLES (5 mL of pure Sodium Laureth Sulfate at 30%). The anti-aging and anti-spot efficacy of topical FBA was also evaluated. The results showed that FBA significantly facilitated a strong skin depigmenting activity on UV and brown spots, increasing firmness and elasticity. These effects have been attributed to butyrate, as the results of skin permeation experiments indicated that FBA did not reach the bloodstream, suggesting that the cosmetic benefits were mediated by butyrate alone [7,9]. Thus, FBA could offer an innovative solution for harnessing the benefits of butyrate in the dermatology field, effectively overcoming the organoleptic limitations associated with this short-chain fatty acid.

In the present study, we explored the properties, mechanisms of action, and potential health benefits of FBA on human keratinocytes, exploring its effects on cell proliferation, differentiation, permeability, oxidative stress, and wound healing. Our results highlighted the beneficial effects of FBA and further confirmed the potential of using this compound in cosmetic preparations.

## 2. Results

### 2.1. FBA Treatment Increases Proliferation of Human Keratinocytes

FBA promoted the proliferation of human keratinocytes, the primary cell type found in the epidermis. This effect is crucial for skin repair, regeneration, and overall skin health. A robust and proliferative keratinocyte population is essential for maintaining the skin’s protective barrier. By promoting keratinocyte growth, FBA helps strengthen the skin barrier, protecting against environmental insults, pathogens, and water loss. When HaCat cells were stimulated at different times (0–48 h) and doses (0.1–1 mM), the results of the MTT assay showed that FBA increased HaCaT cell proliferation, with a maximum effect at 0.1 mM of FBA for 18 h (*p* < 0.05).

### 2.2. FBA Improves Skin Barrier Integrity

**Figure 1:**
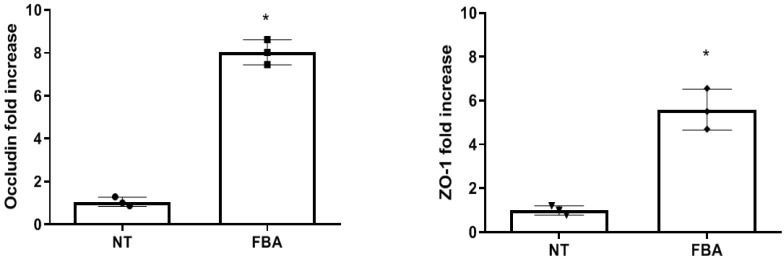
Occludin and ZO-1 gene expression. HaCaT cells show a significant increase in tight junction (occludin and ZO-1) gene expression after treatment with FBA for 18 h at 0.1 mM. Data were analyzed using the one-way ANOVA test. Data are expressed as means ± SDs of three independent experiments, each performed in triplicate. * *p* < 0.0001 vs. NT.

It has been well demonstrated that tight junction (TJ) molecules play a crucial role in the skin’s barrier. Increased keratinocyte proliferation leads to a denser and more cohesive epidermal layer, which is fundamental for maintaining a healthy skin barrier. To determine the beneficial action elicited by FBA on the integrity of the epithelial barrier, occludin and zonula occludens-1 (ZO-1) gene expression was evaluated. As shown in Figure 1, HaCat cells after 7 days of differentiation treated with 0.1 mM FBA show a higher expression of the occludin and ZO-1 genes.

### 2.3. FBA Inhibits Oxidative Stress

**Figure 2:**
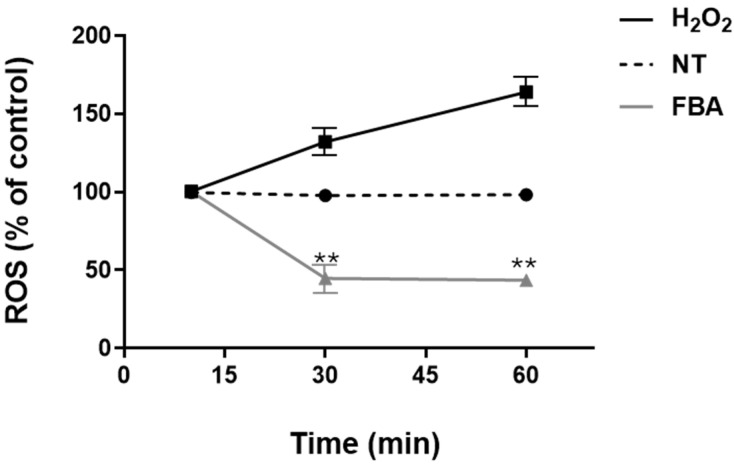
FBA inhibits oxidative stress in HaCaT cells. HaCaT cells were treated with 0.1 mM FBA for 18 h. H_2_O_2_ was used as positive control. FBA induced a significant decrease in ROS production in a time-dependent manner. Data were analyzed using the one-way ANOVA test. Data are expressed as means ± SDs of three independent experiments, each performed in triplicate. ** indicates significant difference in FBA vs. H_2_O_2_ and NT (*p* < 0.001), respectively.

Oxidative stress results from an imbalance between the production of reactive oxygen species (ROS) and the body’s ability to neutralize these harmful molecules with antioxidants. Excessive oxidative stress can lead to cellular damage, inflammation, and skin diseases. We found that 0.1 mM FBA inhibited oxidative stress, as demonstrated by a significant reduction in ROS levels in HaCaT cells (Figure 2).

**Figure 3:**
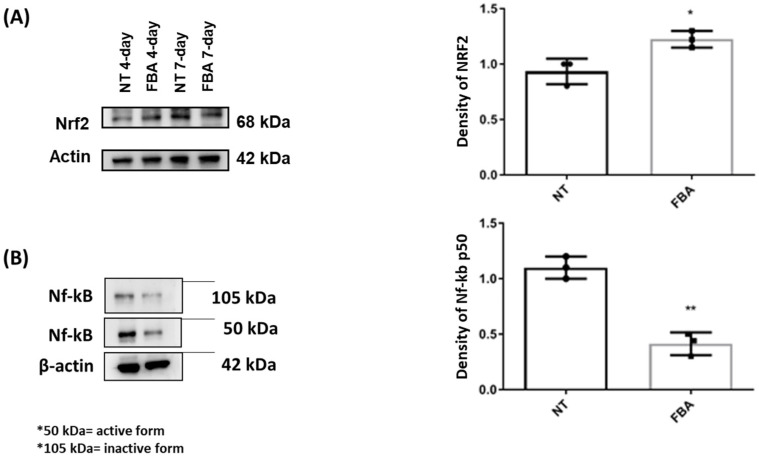
Effects of FBA on Nrf2 and Nf-kB transcription factors. HaCaT cells were treated with FBA for 18 h. Western blot assay of Nrf2 (**A**) and Nf-kB (**B**) was performed on protein extracts of HaCaT cells. Treatment with FBA induced a significantly greater increase in Nrf2 transcription factor than untreated cells (NT) while a significant decrease in Nf-kB was observed. The amounts of these proteins and β-actin were measured by Western blot. Full-length lanes of Western blot are shown in Supplementary Figure S1. The histogram below shows the optical density of the proteins, obtained with Image Lab software 3.0. Data were analyzed using the one-way ANOVA test. Data represent the means ± SDs of three independent experiments, each performed in triplicate. * and ** indicate *p* <  0.05 and *p* < 0.001 vs. NT, respectively.

We also explored if FBA could modulate redox-sensitive signaling pathways that regulate the cellular response to oxidative stress. We found that FBA may influence the Nrf2 (nuclear factor erythroid 2-related factor 2) pathway, which controls the expression of various antioxidants and cytoprotective genes. The transcription factor Nrf2 plays a pivotal role in controlling the expression of antioxidant genes that ultimately exert anti-inflammatory functions. NF-κB is a transcription factor that plays a key role in regulating immune and inflammatory responses. Upon activation, NF-κB translocates to the nucleus and promotes the expression of pro-inflammatory cytokines, chemokines, and adhesion molecules, leading to inflammation. The effect of FBA on Nrf2 and NF-kB transcription factors is shown in Figure 3. Specifically, we demonstrated that FBA elicited a significant increase in the expression of the transcription factor Nrf2 in human keratinocytes (Figure 3A) and significantly reduced the Nf-kB activation (Figure 3B).

### 2.4. FBA Promotes Differentiation of Human Keratinocytes

**Figure 4:**
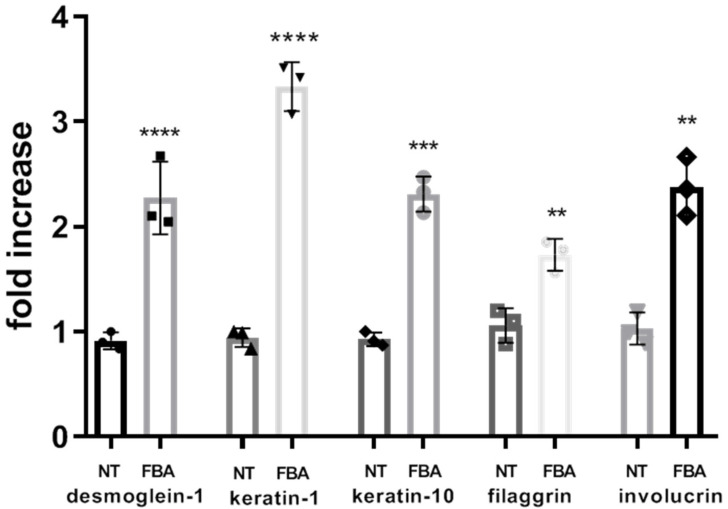
FBA promotes differentiation of human keratinocytes. The expression levels of desmoglein-1, keratin-1 (KRT-1), keratin-10 (KTR-10), filaggrin (FLG), and involucrin were significantly increased in HaCaT cells exposed to 0.1 mM of FBA during 18 h. Real-time PCR analysis was performed using the comparative threshold cycle (CT) method. Gene expression was normalized against reference glucuronidase beta (GUS-B) gene expression. Data are expressed as mean ± SD and were analyzed by pairwise *t*-test. **** *p* < 0.0001 NT-Keratin-1 vs. FBA-Keratin-1 and NT-desmoglein-1 vs. FBA-desmoglein-1; *** *p* < 0.001 NT-keratin-10 vs. FBA-keratin-10; ** *p* < 0.005 NT-filaggrin vs. FBA-filaggrin and NT-involucrin vs. FBA-involucrin.

FBA enhances the synthesis of structural proteins forming the corneocyte envelope and maintaining the structural integrity of the skin barrier. Improved protein synthesis leads to a more resilient and impermeable skin barrier. To test the positive effects of FBA on epidermal keratinocyte differentiation, we evaluated several differentiation markers (desmoglein-1 (DSG-1), keratin-1 (KRT-1), keratin-10 (KTR-10), filaggrin (FLG), and involucrin (IVL) (Figure 4). By stimulating the cells with 0.1 mM FBA for 18 h, we found a significant increase in all markers of differentiation.

### 2.5. FBA Promotes the Expression of the Cellular Matrix Proteins

**Figure 5:**
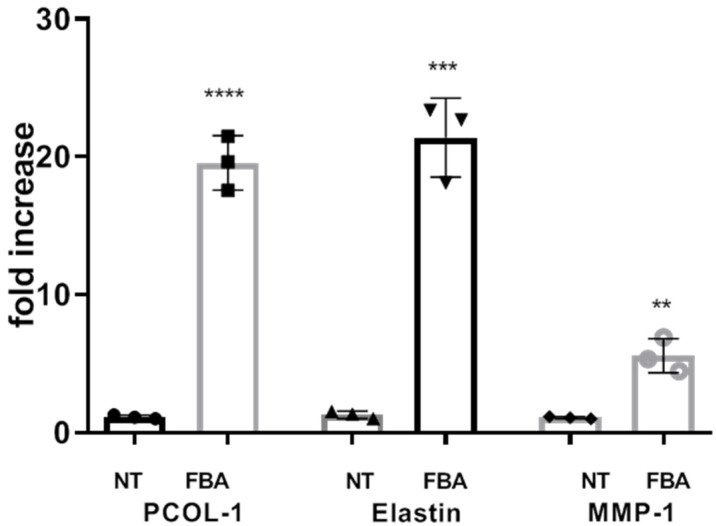
FBA promotes collagen synthesis. HaCaT cells were stimulated with 1 mM FBA for 18 h. The expression levels of pro-alpha chain of collagen type I (PCOL-1), elastin, and MMP-1 were significantly increased in HaCaT cells exposed to FBA. Real-time PCR analysis was performed by the comparative threshold cycle (CT) method. Gene expression was normalized against the expression of the reference glucuronidase beta (GUS-B) gene. Data are expressed as mean ± SD and were analyzed by pairwise *t*-test. **** *p* < 0.0001 NT-PCOL vs. FBA-PCOL, *** *p* < 0.001 NT-Elastin vs. FBA- Elastin, ** *p* < 0.005 NT-MMP-1 vs. FBA-MMP-1.

The extracellular matrix (ECM) is crucial for providing structural support, maintaining skin integrity, and facilitating wound healing. Here, we explored the effects of FBA on the expression of ECM proteins. The pro-alpha chain of collagen type I (PCOL-1), elastin, and MMP-1 of HaCat cells were measured using quantitative RT-PCR. FBA promoted the expression of cellular matrix proteins, as shown in Figure 5.

### 2.6. FBA Facilitates Wound Healing

**Figure 6:**
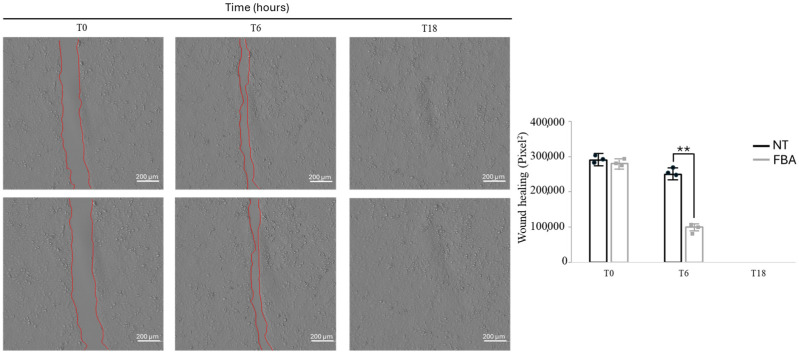
FBA induces wound repair. HaCaT cells were scratched with a sterile 0.2 mL tip. Incubation with 0.1 mM FBA was performed in a serum-free medium. Wound width (red lines) was measured microscopically using software at the beginning of incubation and expressed in pixel. It can be noted that the wound width closes in a shorter time in FBA-treated cells compared to the NT ones. ** *p* < 0.001 NT vs. FBA.

Enhanced keratinocyte proliferation is crucial for an efficient wound healing process. Keratinocytes migrate to the wound site, proliferate, and form new tissue, which is essential for closing wounds and restoring the skin barrier. The effect of 0.1 mM FBA treatment is shown in Figure 6. FBA accelerated the aforementioned processes, leading to faster and more effective wound healing. Specifically, we observed wound closure at different times and doses of FBA and the most effective concentration was 0.1 mM FBA for 18 h (Figure 6). We saw that the wound in the HaCaT cells treated with 0.1 mM of FBA closed in a shorter time than in the untreated cells (Figure 6). This result demonstrates that FBA has healing properties, helping the epidermis to restore its integrity.

### 2.7. Butyrate Concentration Following HaCaT FBA Stimulation

**Figure 7:**
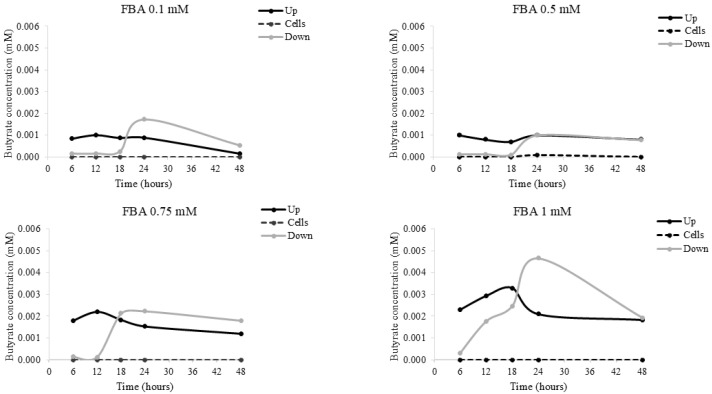
Butyrate concentration in HaCat cells. HaCaT cells were incubated with 0.1–1 mM FBA; following 6–48 h, the liquid above the cells (up), below the cells (down), and in the cell layer (cells) was collected. The butyrate concentration was evaluated by LC-UV detection. Data are expressed as means ± SDs of three independent experiments, each performed in triplicate.

Figure 7 shows butyrate concentration, quantified by GC-MS, in the supernatant above (up) the cells, below (down) the cells, and in the cell layer (cells) of human keratinocytes exposed to FBA. The results showed that butyrate was not present in the cell layer, and lesser amounts were found above and below the supernatants (ranging between 0–0.004 mM).

## 3. Discussion

The benefits of butyrate for skin health are substantial, encompassing anti-inflammatory, barrier-enhancing, wound-healing, antimicrobial, and antioxidant effects [3]. Incorporating butyrate-rich foods, such as dietary fibers that support the gut production of this short-chain fatty acid can contribute to overall skin wellness [10]. As research continues to uncover more about butyrate’s role, it remains a promising component for maintaining and improving skin health. Unfortunately, the main limiting factors for the use of butyrate in dermatology are related to the unfavorable sensory and physicochemical properties. FBA could represent a promising butyrate releaser with a range of potential skin health benefits. Its enhanced stability and bioavailability make it a superior alternative to free butyrate for therapeutic applications. As research continues to uncover the full scope of its effects, this compound holds significant potential for use in managing inflammatory conditions, supporting body health [3]. Previous evidence confirmed that FBA exerts a potent anti-inflammatory action in different experimental models with a similar extent of butyrate [11,12].

To see whether FBA treatment could induce a positive action on epithelial cells, we evaluated the proliferation of HaCat cells. We demonstrated that FBA treatment is able to increase cell proliferation.

TJ are central structures that play critical roles in the barrier function of epithelial cells [13]. The surface-expressed protein occludin is an essential structural molecule of the TJ that regulates barrier permeability. ZO-1, also known as TJ protein-1, is a peripheral membrane protein. It serves as a scaffold protein that connects and anchors TJ filament proteins, which are fibril-like structures within the lipid bilayer, to the actin cytoskeleton [12]. Both occludin and ZO-1 gene expression appear to increase after FBA treatment. Hence, we found that FBA treatment has beneficial effects on the integrity of the keratinocyte monolayer. NF-kB and Nfr2 are key pathways regulating the balance in cellular redox status and responses to stress and inflammation [14]. It has been demonstrated that Nfr2 overexpression inhibited NF-kB activation [15]. FBA effectively inhibits oxidative stress and NF-κB activation, providing significant benefits for skin health. Its antioxidant properties neutralize harmful free radicals, while its ability to block NF-κB activation reduces inflammation. These effects are crucial for preventing and managing inflammatory skin conditions, offering anti-aging benefits, enhancing skin barrier function, promoting wound healing, and maintaining overall skin health.

To evaluate the effects of FBA on skin keratinocyte differentiation and collagen production, we studied some markers involved in these processes. Desmoglein-1 (DSG1), a desmosomal protein, maintains the structure of epidermis through its adhesive function [16]. Keratin 1 (KRT1) and its heterodimer partner keratin 10 (KRT10) are major constituents of the intermediate filament cytoskeleton in suprabasal epidermis [17]. Filaggrin aggregates keratin filaments and promotes cytoskeleton condensation and cell compaction to form the cornified envelope [18]. Involucrin is involved in the formation of the cornified envelope, the cohesion of corneocytes, and the consequent enhancement of skin barrier function [17]. Matrix metalloproteinases (MMPs) are a family of zinc-containing peptide hydrolases that can lead to the degradation of ECM [18]. UV-irradiation induces the production of reactive oxygen species (ROS) that can lead to the activation of MMPs, which degrade the collagen matrix system in the dermis [19,20]. MMP-1 has been reported to lead to collagen degradation due to oxidative stress [19]. Our results show that keratinocytes treated with FBA exhibited a significant increase in proteins involved in differentiation, such as DSG-1, KRT-1, KRT-10, FLG, and IVL, and an increase in cellular matrix proteins involved in collagen production, such as P-COL1, elastin, and MMP-1.

Disruptions of skin homeostasis may be associated with various skin diseases and abnormal healing of skin wounds [21]. The wound healing process is based on a valuable molecular mechanism divided into three different phases: inflammation, cell proliferation, and cell differentiation [22]. The healing process can be hampered in the case of large, long-lasting, and difficult-to-treat wounds. Given the complexity of the wound healing process, it is necessary to develop functional dressing materials that stimulate reparative and regenerative processes and have a positive effect on infected and/or difficult-to-heal wounds [23].

In summary, FBA, through its ability to release butyrate, promotes cell differentiation, inhibits oxidative stress, and enhances membrane integrity. Therefore, it was investigated whether butyrate is present within the cells after FBA treatment. The results showed that butyrate was detected only in the medium beneath the cell layer, suggesting that cells actively utilize butyrate to carry out their functions.

## 4. Materials and Methods

### 4.1. N-(1-Carbamoyl-2-phenyl-ethyl) Butyramide (FBA)

N-(1-carbamoyl-2-phenyl-ethyl) butyramide (FBA) is structurally characterized by the attachment of a butyramide group to a phenylalanine-derived moiety. This modification not only improves the stability of the compound but also enhances its ability to be absorbed and utilized in the body, addressing some of the limitations associated with free butyrate. The biological effects of FBA are largely attributed to its ability to release butyrate upon metabolism and its potential effects on health benefits, as previously reported [10].

N-(1-carbamoyl-2-phenyl-ethyl) butyramide supplied by Blue California, Rancho, Santa Margharita, CA 92688; Lot Number: 20200905 served as the test product. This FBA molecule was confirmed to have >99% purity using high-performance liquid chromatography (HPLC) and was absent from heavy metal (i.e., lead, mercury, arsenic, and cadmium) and microbiological contaminants. The test product was obtained in a powdered form and stored at 15 to 25 °C protected from light.

### 4.2. Human Keratinocyte Cell Line

For all experiments, the human immortalized keratinocyte cell line, HaCaT (American Type Culture Collection, Middlesex, UK; accession number: CVCL-0038), was used. Cells were tested for mycoplasma and confirmed to be free of mycoplasma contamination. Cells were maintained in Dulbecco’s modified Eagle’s medium (DMEM) supplemented with Fetal Bovine Serum (FBS) (Sigma-Aldrich; St Louis, MO, USA) 10%, L-glutamine (Sigma-Aldrich; St. Louis, MO, USA) 1%, and penicillin/streptomycin (Sigma-Aldrich; St. Louis, MO, USA) 1%. The cells were maintained in culture in 75 cm^2^ flasks (Corning Incorporated, Corning, NY, USA) equipped with a porous cap that allows gas exchange, at 37 °C in an atmosphere of 5% CO_2_ and 90% relative humidity. The culture medium was changed every 2 days until reaching confluence. All experiments were performed in triplicate and repeated twice.

### 4.3. Cell Proliferation Assay

HaCaT cell proliferation was assessed using the MTT assay (the bromide salt of 3-(4,5-dimethylthiazol-2-yl)-2,5-diphenyl tetrazolium) (Sigma-Aldrich, Milan, Italy). Briefly, cells (10^4^ cells/well) were seeded in 24-well plates (Corning, Inc., New York, NY, USA) and stimulated at different times (0–48 h) and doses of FBA (0.1–1 mM) at 37 °C in a 5% CO_2_ incubator. For each time and dose, cell viability was evaluated by adding an MTT solution (5 mg/mL) and incubating for 1 h. The medium was then removed, and the converted dye was solubilized with acidic isopropanol (0.04–0.1 N HCl in absolute isopropanol). Absorbance was measured at 570 nm using an Epoch Microplate Spectrophotometer (Bioteck, Winooski, VT, USA).

### 4.4. Cell Stimulation Protocol

After 7 days of differentiation, HaCaT cells were stimulated at different time points (6–12–18–24–30–36–42–48 h) and doses (0.1, 0.5, 0.75, 1 mM) of FBA in a Transwell plate. The supernatant above the cells (up), below the cells (down), and the cell layer (cells) were collected at different time points to obtain butyrate concentrations (by GC-MS). Moreover, the results of these dose–response experiments suggested that 0.1 mM of FBA for 18 h was the most effective experimental condition for all tested variables. Cells with medium alone were used as a negative control. Subsequently, the supernatants were collected and stored at −20 °C for further use. The experiment was performed three times in triplicate.

### 4.5. Butyrate Concentration by Liquid Chromatography Analysis

For liquid chromatography analysis, HaCaT cells were incubated with 0.1–1 mM FBA. After 6–48 h, 300 μL of the liquid above the cells (up), below the cells (down), and in the cell layer (cells) of each sample was collected to demonstrate the role of FBA as a butyrate releaser using high-quality analytical techniques, such as LC-UV and GC-MS. Then, 300 μL of ethyl acetate was added, centrifuged for 5 min at 6000 rpm. Subsequently 10 µL of supernatant was collected, diluted 1: 10,000 in ethanol, and injected into the chromatographic system. Further, 1 mL of ethyl acetate was added to dried cell samples, vortexed for 30 s, and centrifuged for 5 min at 6000 rpm. Subsequently, the supernatant was diluted as described above. The analysis of butyrate concentration was carried out using a chromatographic system consisting of Agilent Technologies 1200 Series (Agilent, Santa Clara, CA, USA). The system was equipped with a 7725 Rheodyne injection valve with a 20 μL loop and an ultraviolet (UV)–visible detector (Shimadzu Model SPD10 AV) set to a wavelength of 200 nm. The analysis was performed using a Phenyl Hexyl column (250 × 4.6 mm, 100 Å) (Kinetex, Torrance, CA, USA) equipped with a 4 × 3.0 mm Precolumn (Phenomenex, CA, USA). Before use, the mobile phase solvents were acetonitrile:distilled water (30:70 *v*/*v*) at flow rate of 0.5 mL/min. The mobile phase solvents were vacuum filtered using 0.45 μm nylon membranes (Millipore, Burlington, MA, USA). FBA quantification was achieved according to a previous validated method [24]. Data acquisition and integration were managed using Cromatoplus 2011 software, and each sample was injected three times to assess the instrument repeatability.

### 4.6. Gas Chromatography–Mass Spectrometry

Samples were analyzed by gas chromatography–mass spectrometry (GC-MS) (GC-7890A, Agilent Technologies; MS-5977A MSD, Agilent Technologies). In brief, 100 µL of each sample was diluted with 900 µL of saline, and 500 µL of this solution was added to 20 µL of H_3_PO_4_ 85% (*w*/*v*) and vortexed for 5 min. Then, 500 µL of diethyl ether was added to each sample. The suspension was vortexed for 5 min and centrifuged at 14,000 rpm for 30 min at room temperature. After that, the supernatant was collected and sodium sulfate anhydrous was added. Finally, the organic phase was placed in a new glass tube for GC–MS analysis. The GC method was programmed to achieve the following run parameters: initial temperature of 90 °C, hold for 2 min, a ramp of 2 °C/min up to a temperature of 100 °C, hold of 10 min, and a further ramp of 5 °C/min up to a final temperature of 110 °C for a total run time of 21 min.

### 4.7. Reactive Oxygen Species Production

Reactive oxygen species (ROS) production was assessed in differentiated HaCaT cells by 7′-dichlorofluorescein diacetate (DCFH-DA) (Sigma-Aldrich) and spectrofluorometer. Briefly, after stimulation with 0.1 mM FBA, DCFH-DA (20 µM) was added to the non-treated and treated cells for 30 min at 37 °C in the dark. After two washes in PBS, intracellular ROS levels were measured in a fluorometer (SFM 25, Kontron Instruments; Tokyo, Japan). As a positive control, hydrogen peroxide (H_2_O_2_) (Sigma-Aldrich) was used at concentrations of 10 mM for 15, 30, and 60 min.

### 4.8. Protein Extraction

For the extraction procedure, the medium was removed, and the cells were washed 2 times with 1X PBS. Then, trypsin was used to detach the cells. They were then centrifuged twice at 13,000 rpm, at 4 °C for 5 min. Then, 200 µL of RIPA lysis buffer (Thermo Fisher Scientific, Waltham, MA, USA) was added. The samples were kept on ice for at least 30 min and subsequently centrifuged at 13,000 rpm, at 4 °C for 30 min. The supernatant was collected at the end. The total protein concentration of each sample was determined by the Bradford test on the SmartSpec Plus UV/Visible spectrophotometer (Bio-Rad, Hercules, CA, USA).

### 4.9. Western Blot Analysis

Western blotting analysis was performed following protein extraction from cells using RIPA buffer (50 mM Tris–Hcl, pH 7.6, 150 mM NaCl, 1 mM MgCl_2_, 1% NP-40) supplemented with a protease and phosphatase inhibitor cocktail. Protein concentrations were determined by using the BioRad protein assay dye reagent, with BSA (PanReac AppliChem, Cranbury, NJ, USA) as the standard. Proteins were separated by SDS–Polyacrylamide gel electrophoresis (SDS-PAGE) at 150 V during 10 min and 190 V during 40 min, and subsequently transferred onto Polyvinylidene fluoride (PVDF) membranes (ImmobilonR-Transfer Membrane, Tullagreen, Carrigtwohill, Co., Cork, Ireland). To block nonspecific protein binding, membranes were incubated for 1 h at room temperature in a solution containing 5% nonfat dry milk (PanReac AppliChem) and 0.2% Tween20/PBS. Membranes were then incubated overnight at 4 °C with primary antibodies targeting Nf-kB p105/p50 (1:1000; Invitrogen, Waltham, MA, USA, 51-3500), Nrf2 (1:1000; Abcam, Cambridge, UK, ab894432), and β-actin (1:5000; Elabscience, Houston, TX, USA, E-AB-20034). The following day, membranes were incubated with the peroxidase-linked (HRP) conjugated anti-rabbit IgG (1:2000; Abcam, ab205718) or anti-mouse IgG (1:5000; ImmunoReagents, Raleigh, NC, USA, GtxMu-003-DHRPX), and protein expression was visualized using an enhanced chemiluminescence detection solution (ECL Wester Antares; Cyanagen, Bologna, Italy). The relative band intensity of each protein was quantified by the normalization to the β-actin loading control, using Image Lab Software 3.0 (Biorad, Hercules, CA, USA).

### 4.10. Quantitative Real-Time PCR

Total RNA was isolated from cells with TRIzol reagent (Gibco BRL, Paisley, UK), quantified using a NanoDrop Spectrophotometer and purity was verified by A260/280 and A260/230 absorbance ratio. RNA was reverse transcribed in cDNA with a High-Capacity RNA-to-cDNATM Kit (Life Technologies, Waltham, MA, USA) according to the manufacturer’s instructions. Complementary DNA (cDNA) was stored at −80 °C until use. Quantitative real-time PCR (qRT-PCR) analysis was performed using Taqman Gene Expression Master Mix (Applied Biosystems, Vilnius, Lithuania) to evaluate the gene expression of occludin (Hs05465837_g1), ZO-1 (Hs03829530_s1), desmoglein-1 (Hs00355084_m1), keratin-1(Hs00196158_m1), keratin-10 (Hs00166289), filaggrin (Hs00856927), involucrin (Hs00846307_s1), PCOL-1 (Hs00241807_m1), elastin (Hs00230757_m1), and MMP-1 (Hs00899658_m1). The TaqMan probes for these genes were inventoried and tested at the Applied Biosystems manufacturing facility (QC). The amplification protocol was 40 cycles of 15 s of denaturation at 95 °C, 60 s of annealing at 60 °C, and 60 s of elongation at 60 °C in a Light Cycler 7900HT (Applied Biosystems, Grand Island, NY, USA). Data were analyzed using the comparative threshold cycle method. We used the beta-glucuronidase (GUSB) gene as the housekeeping gene (forward primer: 5′-GAAAATATGTGGTTGGAGAGCTCATT-3′; reverse primer: 5′-CCGAGTGAAGATCCCCTTTTTA-3) to normalize the level of mRNA expression.

### 4.11. Scratch Wound Healing Assay

The scratch wound healing assay was performed as previously described [Optimized Scratch Assay for In Vitro Testing of Cell Migration with an Automated Optical Camera; Michelle Vang Mouritzen, Havard Jenssen] with some modifications. Briefly, HaCaT cells were seeded into a 6-well plate at a density of 5 × 10^5^ cells/well in Dulbecco’s modified Eagle’s medium (DMEM) supplemented with 10% fetal bovine serum (FBS), 100 units/mL penicillin, and 100 μg/mL streptomycin. The culture plate surface was pre-coated using a poly-L-lysine solution. Cultures were maintained at 37 °C in a humidified atmosphere containing 5% CO_2_. Once the cells reached 95% confluence, they were treated with 10 µg/mL of mitomycin C, a DNA synthesis inhibitor, for 2 h at 37 °C, in an appropriate volume of medium, and washed with PBS before scratching. A scratch was then created using a 200 μL pipette tip, followed by treatment with 1 mM FBA.

Wound healing was monitored over 18 h using an inverted microscope (Zeiss Celldiscoverer 7, Oberkochen, Germany) with a 10× objective lens and quantified using the Zen blu 3.0 software.

### 4.12. Statistical Analysis

The Kolmogorov–Smirnov test was used to determine whether variables were normally distributed. Data were analyzed using paired *t*-test and compared by one-way ANOVA test, followed by Tukey post hoc test. The level of significance for all statistical tests was two-sided, *p*  <  0.05. All data were collected in a dedicated database and analyzed by a statistician using GraphPad Prism 7 (La Jolla, CA, USA).

## 5. Conclusions

Our results highlighted that FBA positively modulates cell differentiation and proliferation, improves barrier function, protects against oxidative stress, and has a beneficial effect on skin repair, promoting wound closure. Therefore, FBA represents an innovative method to harness the benefits of butyric acid in cosmetics, overcoming the organoleptic limitations of the latter. Future studies should also investigate the potential synergistic effects of FBA with other skin-beneficial compounds, as well as its stability and efficacy in complex formulations. Furthermore, reduced butyrate production plays a significant role in atopy, perpetuating inflammation and impairing skin barrier function. This suggests that FBA could have potential cosmetic applications for the treatment of atopic dermatitis, improving skin barrier function and reducing inflammation [24]. Further research should investigate its clinical impact in atopic dermatitis models and test the efficacy of FBA-based products in therapeutic and preventive applications.

## Data Availability

Data are contained within the article.

## Supplementary Materials

The following supporting information can be downloaded at: https://www.mdpi.com/article/10.3390/ph18040517/s1, Figure S1: Full length Western Blot images.
