# Supplementary figures and images for: The Beneficial Effects of a N-(1-Carbamoyl-2-phenyl-ethyl) Butyramide on Human Keratinocytes
Source: Pharmaceuticals (Basel). 2025 Apr 1;18(4):517. doi: 10.3390/ph18040517 (PMC12030237; doi:10.3390/ph18040517)

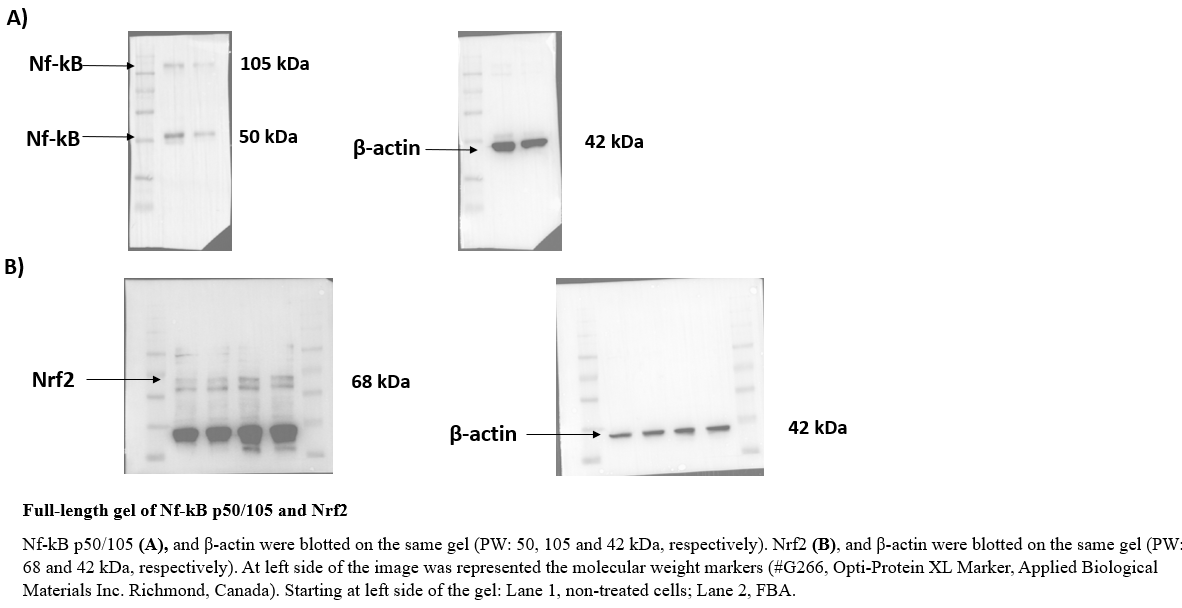

Supplement: Supplementary file 1 [file pharmaceuticals-18-00517-s001.zip › pharmaceuticals-3476301-supplementary.png]
